# Recent advances in the biology and therapy of medullary thyroid carcinoma

**DOI:** 10.12688/f1000research.12645.1

**Published:** 2017-12-28

**Authors:** Barry Nelkin

**Affiliations:** 1 Sidney Kimmel Comprehensive Cancer Center, Johns Hopkins University School of Medicine, 1650 Orleans Street, Baltimore, MD, 21287, USA

**Keywords:** Medullary thyroid cancer, RET, Treatment, RET inhibitors, RAS inhibitors

## Abstract

Medullary thyroid cancer (MTC) is a relatively uncommon yet prognostically significant thyroid cancer. Several recent advances in the biology and current or potential treatment of MTC are notable. These include a new understanding of the developmental biology of the thyroid C cell, which heretofore was thought to develop from the neural crest. RET, encoded by the most common driver gene in MTC, has been shown to be a dual function kinase, thus expanding its potential substrate repertoire. Promising new therapeutic developments are occurring; many have recently progressed to clinical development. There are new insights into RET inhibitor therapy for MTC. New strategies are being developed to inhibit the RAS proteins, which are potential therapeutic targets in MTC. Potential emerging immunotherapies for MTC are discussed. However, gaps in our knowledge of the basic biology of the C cell, its transformation to MTC, and the mechanisms of resistance to therapy impede progress; further research in these areas would have a substantial impact on the field.

## Introduction

Medullary thyroid cancer (MTC) is a relatively uncommon (about 1,400 new cases per year in the US) cancer of the thyroid C cells, yet accounts for a substantial fraction of thyroid cancer mortality. Excellent comprehensive reviews of the biology, genetics, and management of MTC have been published ^1– 9^. Briefly, in 25% of MTC cases, the disease is hereditary, occurring as part of the MEN 2 syndromes (MEN 2A, MEN 2B, and familial medullary thyroid cancer) due to germline activating mutations of the *RET* receptor tyrosine kinase gene. *RET* is also mutated in about 50% of sporadic cases of MTC; in both hereditary and sporadic cases, specific mutations are correlated with phenotype and prognosis ^4, 6^. MTC is resistant to cytotoxic chemotherapy. RET inhibitors have provided significant clinical benefit; two RET inhibitors, vandetanib and cabozantinib, are US Food and Drug Administration (FDA)-approved for the treatment of advanced MTC ^10– 12^. In this short and necessarily selective review, I discuss some recent advances in MTC and in related areas that are likely to affect MTC. I have tried to point out some other areas in which MTC research might be productively focused.

## Biology of medullary thyroid cancer

### Medullary thyroid cancer is not derived from the neural crest

For almost half a century, thyroid C cells have been thought to be derived from the neural crest. This hypothesis was based largely on chick-quail xenotransplantation studies by Le Douarin *et al*. ^13, 14^, which revealed that avian calcitonin-producing ultimobranchial bodies were derived from the neural crest. This model was extrapolated to mammalian species, in which the ultimobranchial bodies are derived from the fourth pharyngeal pouch and invade the thyroid during development, differentiating into C cells. The neural crest derivation of mammalian ultimobranchial bodies and thyroid C cells was challenged by Kameda *et al*. ^15^, who showed by cell-lineage tracing that mouse ultimobranchial bodies were not derived from neural crest cells. Recently, Johansson *et al*. ^16^ used an elegant lineage-tracing scheme to confirm that mouse ultimobranchial bodies and thyroid C cells are not derived from neural crest cells but rather from pharyngeal endoderm. Nevertheless, C-cell development is significantly influenced by neural crest-derived cells ^17^. These findings may have an impact on our understanding of the biology of mixed histology tumors, as discussed by DeLellis and Mangray ^8^. While many of these are likely to be “collision tumors”, the endodermal origin of both C cells and follicular cells strengthens the possibility that some of these tumors arise from a single progenitor cell.

### Understanding RET

A potential dramatic change in our understanding of RET kinase function is underway. Bagheri-Yarmand *et al*. ^18^ reported that RET is not only a membrane-bound receptor tyrosine kinase, but also is localized to the nucleus. There, one of its functions is to inhibit the pro-apoptotic transcription factor ATF4. This antagonism of ATF4 activity provides a potential mechanism for the anti-apoptotic function of RET ^19^. Remarkably, Bagheri-Yarmand *et al*. ^18^ showed that RET appears to accomplish this inhibition by *threonine* phosphorylation of ATF4, marking it for proteolytic degradation; thus, RET is a dual specificity tyrosine-threonine kinase. Plaza-Menacho *et al*. ^20^ recently confirmed and extended this finding of dual specificity, showing that RET is autophosphorylated at serine 909 in the activation domain. While they show that this phosphorylation has no effect on RET enzymatic activity *in vitro*, they showed that it affects RET signaling in an *in vivo* model. In the *Drosophila* Ret2B model of MEN 2B, *dRet* M955T mutation, analogous to human *RET* M918T, results in a rough eye phenotype ^21^. When a further *dRet* S946A mutation (analogous to human S909A) was introduced, the rough eye phenotype was rescued. Plaza-Menacho *et al*. ^20^ speculate that RET pS909 may function in docking of downstream effector proteins. This expanded specificity has the potential to expand the universe of substrates of RET; in any case, important RET effector substrate proteins still await identification.

### Under-researched area: biology of C-cell transformation

Thus far, relatively little is known regarding the molecular steps of C-cell transformation leading to MTC ^22^. This is due in large part to the rarity of C cells in the normal human and mouse thyroid, limiting their study to *in situ* methods. Cell-sorting methods applied to thyroid glands ^23– 25^ suggest that isolation of small numbers of normal or early dysplastic C cells may be feasible; functional and “omics” studies on such cell populations could sharply address our dearth of knowledge in this important area.

## Genomics of medullary thyroid cancer

MTC cases typically have few mutations ^26^. As mentioned, about 50% of sporadic cases have an activating point mutation in the *RET* gene. The only other common mutations identified in sporadic MTC are *KRAS* and *HRAS*; *NRAS* mutations are infrequent ^26– 31^. Recent reports have described *ALK* and *RET* gene rearrangements in sporadic MTC ^32, 33^. While these rearrangements are infrequent, they indicate the potential for targeted therapy in specific cases; perhaps even more importantly, they suggest the important role that these and other rearrangements may play in MTC. Rearrangements would not likely have been detected in whole exome sequencing studies of MTC ^26^ and can play a dominant driver role in cancer development ^34– 36^.

## Treatment of medullary thyroid cancer

### RET inhibitors: fulfilling the promise

***Introduction.*** Vandetanib and cabozantinib have changed the face of MTC systemic therapy, offering an effective targeted treatment. Nevertheless, both drugs are effective in less than half of patients with MTC and have significant adverse effects, and (even in responsive cases) resistance develops, typically within a few years.

*Adverse effects*. It is not clear which effects are off-target (non-RET) and which are on-target (due to RET inhibition). Since both vandetanib and cabozantinib are multikinase inhibitors, there is some thought that more specific inhibitors may alleviate these effects. However, it is necessary to consider the possibility that the inhibition of some of the other kinases targeted by these drugs contributes to their efficacy. The response of *RET* wild-type (wt) tumors may suggest that this occurs; the ability of both vandetanib and cabozantinib to inhibit VEGFR2 suggests it as a potential candidate target, in which case VEGFR2-dependent adverse effects may be inseparable from efficacy.

*Resistance*. As mentioned above, MTC commonly exhibits intrinsic resistance to RET inhibitors, and even those cases that are initially sensitive to RET inhibitors almost always develop acquired resistance, resulting in disease progression. A critical need in the field is an understanding of the mechanisms of intrinsic and acquired resistance to RET inhibitors in MTC. Numerous mechanisms for resistance to tyrosine kinase inhibitors (TKIs) have been described, including feedback signaling response, secondary mutations, or gene amplification in the TKI, bypass activation of other signaling or survival pathways, and pharmacokinetic/pharmacodynamic (PK/PD) issues; several resistance mechanisms can coexist in a single resistant tumor ^37^. Mathematical modeling of resistance in other cancers has shown that even apparently acquired resistance may exist intrinsically as subclonal cell populations within the tumor, and such resistant cells can be selected by treatment ^38– 40^. That an understanding of the mechanisms of resistance can direct subsequent effective therapeutic strategies is not only intuitive; it has been demonstrated dramatically in many instances for TKIs and other targeted therapeutics ^41– 46^.

Some information regarding resistance to vandetanib and cabozantinib is available, but much further study is urgently needed. Both inhibitors perform poorly on *RET* mutants with V804M or V804L mutations ^47, 48^; these mutations are orthologous to the *BCR-ABL* T315I “gatekeeper” mutation which confers resistance to imatinib in chronic myelogenous leukemia ^49^. A recent article identified *RET* I788N as another mutation conferring resistance to cabozantinib and vandetanib ^50^; I788 is an ortholog of the V654 residue in c- *KIT*, mutation of which confers resistance to imatinib ^51^. PK studies of vandetanib and cabozantinib have been extensive. There is limited bioavailability *in vivo* and this is due in part to substantial plasma protein binding (92–94% for vandetanib ^52^ and more than 99.7% for cabozantinib ^53, 54^). This suggests that resistance may be due in part to limited drug exposure within the tumor. Unfortunately, PD studies in patients treated with these compounds, pivotal for our understanding, have not been reported.

Remarkably, changes in MTC seen on progression after TKI therapy have not yet been reported. Such studies have been uniquely informative in identifying mechanisms of resistance to other therapeutics ^41– 46^. In such studies, progression biopsies are compared with pretreatment samples by using next-generation sequencing to look for secondary mutations, amplifications, and other genomic changes, transcriptomic studies such as RNA-seq, or proteomic studies to look for potential gene expression changes and pathway activation that may account for acquired resistance. Given the experience with disease progression on current TKIs and the facile availability of these technologies, one would hope that these elucidating correlative studies soon become standard.

***Cabozantinib: who benefits?*** In the Efficacy of XL184 (Cabozantinib) in Advanced Medullary Thyroid Cancer (EXAM) phase 3 clinical trial of cabozantinib ^12^, MTC patients receiving the drug had a significantly longer progression-free survival (PFS) than patients receiving the placebo control (median PFS 49 versus 17 weeks; hazard ratio [HR] = 0.28, *p* <0.0001). Recent correlative subgroup analysis indicated that patients with a *RET* M918T mutation significantly benefited from cabozantinib treatment ^55, 56^. All other mutational subgroups ( *RET* non-M918T mutation, RAS mutation, RAS wt, *RET* and RAS wt, *RET* unknown status, and *RET* mutation of unknown significance) also exhibited decreased HR in response to cabozantinib treatment; the PFS differences in these subgroups did not reach statistical significance, although in some cases this was likely due to the small size of the subgroups. As the authors note, the effects of cabozantinib in these smaller subgroups will need to be resolved in future prospective studies. Notably, the M918T subgroup was the only group to exhibit a significant increase in overall survival (OS) (44.3 versus 18.9 months; HR = 0.60, *p* <0.03). Thus, it is clear that cabozantinib is effective for MTC patients with the *RET* M918T mutation, but in no case is it yet possible to exclude patients from cabozantinib treatment on the basis of mutation status, especially given the paucity of effective MTC treatments other than RET multikinase inhibitors.

If, as suggested by the correlative data in the EXAM trial, MTC with *RET* M918T mutations derives more clinical benefit from cabozantinib than do other MTC subgroups, what might be the mechanism? Here, our understanding is impeded by our limited knowledge of the biology of *RET* mutations. A few transcriptomic studies have compared MTC cases with *RET* M918T mutations versus other *RET* mutations ^57– 60^, but further work is necessary, since some of the studies were underpowered and consensus among other studies was lacking. One potentially important difference between M918T and other *RET* mutations is in substrate specificity. RET M918T has a relaxed and altered substrate preference ^61^. This is due to destabilization of the substrate recognition domain, which also leads to increased kinase activity ^62– 64^. Speculatively, this altered kinase activity could lead to greater oncogene addiction, which would result in augmented clinical activity of RET inhibitors.

By what mechanism might cabozantinib provide benefit to MTC cases without RET mutations? The potential for other kinase targets of cabozantinib (including VEGFR2, MET, TIE2, RON, and others) ^65^ to contribute to angiogenesis ^65^, malignant transformation ^66^, or other MTC functions has been discussed ^55^. The possibility also exists that wt RET is important in the biology of MTC.

***New RET inhibitors.*** Several new RET inhibitors have been reported ^67^. Some of these compounds have been designed for increased potency, increased specificity (especially as compared with KDR inhibition), improved PKs, or the ability to inhibit RET gatekeeper mutants. The following four interesting compounds are currently in clinical trials.

Alectinib (Roche) is an ALK inhibitor approved for ALK-rearranged non-small cell lung cancer. It is highly specific, inhibiting little other than ALK and RET (concentration resulting in inhibition of 50% of activity [IC _50_] for RET = 4.8 nM) ^68^. At somewhat higher concentrations, alectinib also effectively targets the RET gatekeeper mutants, V804L (32 nM) and V804M (53 nM). While alectinib effectively reduced phospho-RET levels in MTC cell (TT) xenografts, it had little effect on MTC xenograft growth ^68^. This suggests that another target, in addition to RET, may serve to maintain growth in MTC cells. Whether this intrinsic resistance will be seen clinically in MTC is unknown but should be determined shortly; a phase 1 trial for alectinib in MTC and other RET-driven thyroid and lung cancers (NCT03131206) is ongoing.

BLU-667 (Blueprint Medicines) inhibits RET at subnanomolar concentrations ^69^. It efficiently inhibits the RET V804M gatekeeper mutant at similar concentrations and has 70-fold specificity versus KDR. It is in a phase 1 clinical trial (NCT03037385) for cancers with activating *RET* gene alterations, including MTC.

LOXO-292 (Loxo Oncology) inhibits RET at nanomolar concentrations ^70^. It also inhibits the RET gatekeeper mutant at similar concentrations. Importantly, LOXO-292 has been shown to be very specific for RET in kinase assays. Its efficacy in inhibiting the growth of MTC cell xenografts further confirms that targeting RET alone is a viable therapeutic strategy in MTC. LOXO-292 is in a phase 1 clinical trial (NCT03157128) for cancers with activating *RET* gene alterations, including MTC.

RXDX-105 (Ignyta) was originally developed as a BRAF inhibitor (IC _50_ = 14 nM) ^71^. It also efficiently inhibits wt RET (IC _50_ = 1.5 nM) ^71^. This suggests the potential for RXDX-105 to act as a combination treatment, targeting RET and the RAF-MEK-ERK pathway, a combination which may be synergistic in MTC cells ( 72 and unpublished results). RXDX-105 is reported to have 96–100% bioavailability ^71^, a substantial improvement over other RET inhibitors. However, while RXDX-105 does not inhibit KDR, it is a broad-specificity inhibitor ^71^, so it may have significant adverse effects. Moreover, since it does not have activity against CRAF, one can envision “paradoxical activation” of the RAF-MEK-ERK pathway ^73– 75^. Nevertheless, in an ongoing phase 1 trial of RXDX-105 for RET-driven solid tumors (NCT01877811), a partial response was noted in a lung adenocarcinoma patient with a RET gene rearrangement ^76^.

### RAS as a therapeutic target in medullary thyroid cancer

The discovery of RAS mutations in MTC ^26– 31^ highlighted the possibility of targeting RAS therapeutically in some cases of MTC; since RET signaling functions in part through RAS activation ^77^, RAS inhibition also may be a potential therapeutic strategy in MTC with RET mutations. However, RAS has a long history as an intractable target ^78– 80^. It was realized early that targeting RAS-guanosine triphosphate (RAS-GTP) interaction would be difficult since GTP has picomolar affinity for RAS while GTP is present at millimolar concentrations in cells ^81^. A substantial effort to block RAS attachment to the cell membrane by blocking farnesyltransferase was unsuccessful because of alternative cellular mechanisms (geranylgeranylation) of membrane attachment ^78, 79, 82– 84^. Blocking RAS downstream signaling has been attractive but has commonly suffered from ineffectiveness of blocking one pathway and toxicity of blocking multiple pathways ^85^. In recent years, with advances in drug and protein biochemistry, there has been renewed interest and preclinical success in directly inhibiting RAS via a multitude of novel strategies, including small-molecule binding, inhibition of protein interactions, and antisense approaches ^86– 111^. A review of this rapidly advancing field, other than the several examples below, is outside the scope of this review, and the reader is directed to the cited references. In addition, it must be noted that some of the promising therapeutic approaches target specific RAS mutations; at least one of these targeted mutations, KRAS G12C ^87, 91, 92, 95, 100^, is rarely found in MTC ^31^.

***PDE6δ inhibitors.*** The prenyl-binding chaperone protein PDE6δ is necessary for RAS membrane localization ^111^. Early PDE6δ inhibitors, including deltarasin and deltazinone, had low nanomolar affinity for PDE6δ but required micromolar concentrations for RAS inhibitory activity in intact cells ^88, 99^. This discrepancy was recently reported to be due to inhibitor release mediated by the release factor Arl2 ^104^. Newly designed PDE6δ inhibitors with subnanomolar affinity have been shown to be effective in cells and can block KRAS-dependent cell proliferation ^104^. A third generation of highly specific PDE6δ inhibitors covalently binds the active site of PDE6δ, rendering these compounds refractory to Arl2-mediated release ^105^.

***Farnesyltransferase inhibition.*** As mentioned above, the farnesyltransferase inhibitors (FTIs) failed because of alternative prenylation of RAS proteins. However, *HRAS*, the most commonly mutated RAS gene in MTC, cannot employ this alternative prenylation strategy and is dependent upon farnesyltransferase for activity. Thus, HRAS-driven cancers should be sensitive to FTIs. This hypothesis is being tested in a phase 2 study of the FTI tipifarnib for HRAS mutant thyroid or head and neck cancers (NCT02383927). Interestingly, a phase 1 clinical trial of a combination of the FTI tipifarnib and the multikinase (including RET) inhibitor sorafenib showed significant activity for MTC with or without RET mutations; HRAS mutation status was not evaluated ^112^. While the response rate was greater than that seen in another trial employing sorafenib alone ^113^, the trials were not powered for statistical comparison. If this combination is effective, it may be due to additional targets of tipifarnib ^112^, or to synergistic pathway inhibition by tipifarnib and sorafenib, similar to the synergy reported in studies of sorafenib and the MEK inhibitor selumetinib in MTC cells *in vitro* ^72^.

***Farnesyltransferase-mediated delivery of covalent RAS inhibitors.*** In this recently reported, very novel approach, endogenous farnesyltransferase activity was hijacked to mislocalize KRAS by blocking normal prenylation mediated by both farnesyltransferase and geranylgeranyltransferase ^106^. Thus, a farnesyltransferase neosubstrate was designed that binds covalently to the RAS CAAX moiety yet does not promote membrane localization. In cell culture, treatment with this neosubstrate resulted in decreased RAS signaling. The effects were modest, and specificity for transformed cells and efficacy *in vivo* were not addressed. Nevertheless, this report represents the first step in the development of a promising strategy to overcome the ability of KRAS to evade the therapeutic effects of prenylation inhibitors.

***Inhibition of RAS-effector interaction.*** RAS activates its downstream effectors by interaction with their RAS-binding domains (RBDs). Many of the small-molecule RAS direct inhibitors in development appear to work by disrupting these interactions ^97, 98, 103^. Notably, rigosertib, a kinase inhibitor already in phase 3 clinical development for myelodysplastic syndrome, was recently shown to be a RAS mimetic, binding to the RBDs of RAF, PI3K, and RAL-GEF, preventing interaction with RAS and pathway activation ^94^. Rigosertib was shown to inhibit tumorigenesis in several RAS-driven xenograft models.

***Antisense oligonucleotides.*** While therapeutic antisense technology has been hampered by delivery issues, a recent article showed that KRAS expression can be efficiently silenced *in vivo* by systemic treatment with modified 2',4'-constrained ethyl antisense oligonucleotides ^107^; toxicity was not seen, and xenograft growth inhibition was demonstrated. If successful in further studies, such an approach may also be applicable to *KRAS*, *HRAS*, and *RET* mutations in MTC.

### Immunotherapy for medullary thyroid cancer

Exciting recent advances have been made in immunotherapy for a variety of cancers ^114– 118^. The current state of immunotherapy for thyroid cancer, including MTC, was reviewed recently ^119^. While effective immunotherapy has not yet reached MTC, there are several points of interest.

*Immune checkpoint therapy*. It is not clear whether immune checkpoint therapy has promise in MTC. As noted, MTC has a low mutation burden ^26^. MTC also has very low expression of the immune checkpoint ligand PD-L1 ^120^; both of these correlate with poor response to checkpoint blockade ^118, 121, 122^. Nevertheless, in early preclinical and clinical studies, MTC cell or calcitonin vaccines elicited a T-cell response, with apparent antitumor activity ^123– 126^, suggesting the possibility that checkpoint blockade, perhaps in combination with a vaccine, may be effective. A phase 2 clinical trial (NCT03072160) employing the PD-1 checkpoint-blocking antibody pembrolizumab for MTC will begin to explore this potential therapeutic strategy.

*Adoptive cell therapies: tumor-infiltrating lymphocyte, T-cell receptor, and chimeric antigen receptor transfer* ^127– 130^. The low mutation burden of MTC renders these exciting strategies somewhat challenging. Analysis of the most common RET mutations (M918T, C634 mutations) using NetMHC 3.4 software ^131^ failed to identify neoepitopes for avid major histocompatibility complex (MHC) binding in the most common serotypes (unpublished data). Wild-type (and mutated) RET have several epitopes predicted to bind avidly to common MHC alleles; while these could be targeted as potential tumor-associated antigens, the expression of RET in normal cells throughout the body ^132, 133^ raises significant safety concerns of off-tumor toxicity ^134, 135^.

Wang *et al*. reported recently that peptides surrounding the commonly mutated KRAS G12 position are avidly bound and presented by the HLA-A11*01 serotype ^136^, facilitating recognition of these KRAS neoepitopes by T-cell receptors. Wang *et al*. demonstrated that KRAS G12V and KRAS G12D could be recognized by T cells. They isolated high-affinity T-cell receptors specific for these neoepitopes and showed that adoptive transfer of peripheral blood lymphocytes transduced with these KRAS-specific T-cell receptors could effect an antitumor immune response against xenografts harboring the cognate KRAS mutation. While this epitope is not presented by most other common MHC serotypes, Wang *et al*. note that HLA-A11*01 is present in 14% of the U.S. Caucasian population and in 40% of residents of southern China. Moreover, HRAS and NRAS are identical with KRAS in this region, so this strategy could be effective for all RAS isoforms. A phase 1 clinical trial has been opened for HLA-A11*01-positive patients with tumors harboring a KRAS G12V mutation (NCT03190941); this trial is open to MTC patients.

*Antibody-drug conjugates*. MTC and other neuroendocrine tumors commonly express DLL3, a Notch ligand, on their cell surface ^137, 138^. A DLL3 antibody linked to a DNA crosslinker warhead (Rovalpituzumab tesirine; Rova-T) was shown to efficiently inhibit the growth of DLL3-expressing xenografts; notably, tumor-initiating cells were also targeted. A phase 1 trial of Rova-T in small cell lung cancer showed significant clinical activity, almost exclusively in DLL3-highly expressing tumors ^139^. A phase 2 trial of Rova-T in DLL3-expressing solid tumors, including a cohort for MTC, is now open (NCT02709889).

*Intracellular antibodies*. In general, antibody targeting of intracellular targets has been challenging. However, a recent report describes the construction and preclinical use of a monoclonal antibody that penetrates the cytoplasm and targets activated RAS ^140^. The antibody was modified to achieve cellular uptake by endocytosis, followed by endosomal escape into the cytosol. By binding activated RAS, the antibody disrupted downstream signaling pathways and interfered with cell growth. While activity *in vivo* was modest, several strategies were discussed to increase *in vivo* activity. If successful, this promising, albeit nascent, strategy may be applicable not only for RAS but also for targeting other intracellular targets, including RET M918T in MTC.

### Peptide receptor radionuclide therapy

Neuroendocrine cancers, including MTC, frequently express somatostatin receptors (SSTRs), and the potential use of somatostatin analogs for imaging, therapy, or therapeutic targeting has been a common focus for many years. In a recently reported phase 3 trial, midgut neuroendocrine tumors were successfully treated by using peptide receptor radionuclide therapy ( *PRRT*) ^141^. Tumors were stratified by ^111^In-DOTATATE scintigraphy for SSTR expression; in SSTR-positive midgut neuroendocrine tumors (NETs), ^177^Lu-DOTATATE (Lutathera) treatment resulted in highly significant prolongation of PFS and OS and modestly increased objective response rate (ORR). These results have led to European approval of Lutathera for the treatment of midgut NETs; a new drug application has been submitted to the FDA. Could this PRRT strategy work in MTC? In five small studies in MTC, using either ^90^Y- or ^177^Lu- somatostatin analogs ^142– 146^ (reviewed in ^147^), a modest ORR and frequent stable disease were seen. One might envision PRRT as a salvage therapy, stratifying MTC patients with ^68^Ga-SST analog positron emission tomography (PET) ( ^68^Ga-SST analog PET is more sensitive than ^111^In-DOTATATE scintigraphy and detects lesions in about 70% of MTC patients with advanced or recurrent disease ^147– 151^). One concern regarding the potential use of PRRT for MTC is that SSTR expression in MTC has been reported to be focal rather than uniform ^151, 152^; this tumor heterogeneity could significantly limit efficacy.

## Conclusions

The array of exciting directions for advancing our understanding of MTC, and especially for achieving more effective therapies, has never been more promising. Nevertheless, it must be emphasized that further advances will require careful design of basic, translational, and clinical research. Preclinical resources, including cell lines, animal models, and patient-derived xenografts, are currently very limited. A better understanding of the biology of the C cell and its transformation to MTC is critical. As discussed above, it is imperative to understand the mechanisms of MTC progression on therapy, and this will require extensive analysis of progression biopsies. The recent and future advances in the field validate the tireless and ingenious effort that so many researchers have devoted.

## Abbreviations

EXAM, Efficacy of XL184 (Cabozantinib) in Advanced Medullary Thyroid Cancer; FDA, US Food and Drug Administration; FTI, farnesyltransferase inhibitor; GTP, guanosine triphosphate; HR, hazard ratio; IC _50_, concentration resulting in inhibition of 50% of activity; MEN, multiple endocrine neoplasia; MHC, major histocompatibility complex; MTC, medullary thyroid cancer; NET, neuroendocrine tumor; ORR, objective response rate; OS, overall survival; PD, pharmacodynamic; PET, positron emission tomography; PFS, progression-free survival; PK, pharmacokinetic; PRRT, peptide receptor radionuclide therapy; RBD, RAS-binding domain; SST, somatostatin; SSTR, somatostatin receptor; TKI, tyrosine kinase inhibitor; wt, wild-type.

## References

[1] Wells SA Jr Pacini F Robinson BG: Multiple endocrine neoplasia type 2 and familial medullary thyroid carcinoma: an update. *J Clin Endocrinol Metab.* 2013;98(8):3149–64. 10.1210/jc.2013-1204 23744408 PMC5399478

[2] Matias-Guiu X De Lellis R: Medullary thyroid carcinoma: a 25-year perspective. *Endocr Pathol.* 2014;25(1):21–9. 10.1007/s12022-013-9287-2 24343523

[3] Raue F(Ed.): Medullary thyroid carcinoma. Biology, management, treatment. London: Springer.2015;249 10.1007/978-3-319-22542-5

[4] Wells SA Jr Asa SL Dralle H: Revised American Thyroid Association guidelines for the management of medullary thyroid carcinoma. *Thyroid.* 2015;25(6):567–610. 10.1089/thy.2014.0335 25810047 PMC4490627

[5] Hadoux J Pacini F Tuttle RM: Management of advanced medullary thyroid cancer. *Lancet Diabetes Endocrinol.* 2016;4(1):64–71. 10.1016/S2213-8587(15)00337-X 26608066

[6] Romei C Ciampi R Elisei R: A comprehensive overview of the role of the *RET* proto-oncogene in thyroid carcinoma. *Nat Rev Endocrinol.* 2016;12(4):192–202. 10.1038/nrendo.2016.11 26868437

[7] Wang TS Evans DB(Eds.): Medullary thyroid cancer. London: Springer.2016;197 10.1007/978-3-319-39412-1

[8] DeLellis RA Mangray S: Medullary thyroid carcinoma: a contemporary perspective. *AJSP-Reviews and Reports.* 2017;22(4):196–208. Reference Source

[9] Maia AL Wajner SM Vargas CV: Advances and controversies in the management of medullary thyroid carcinoma. *Curr Opin Oncol.* 2017;29(1):25–32. 10.1097/CCO.0000000000000340 27792051

[10] Thornton K Kim G Maher VE: Vandetanib for the treatment of symptomatic or progressive medullary thyroid cancer in patients with unresectable locally advanced or metastatic disease: U.S. Food and Drug Administration drug approval summary. *Clin Cancer Res.* 2012;18(14):3722–30. 10.1158/1078-0432.CCR-12-0411 22665903

[11] Wells SA Jr Robinson BG Gagel RF: Vandetanib in patients with locally advanced or metastatic medullary thyroid cancer: a randomized, double-blind phase III trial. *J Clin Oncol.* 2012;30(2):134–41. 10.1200/JCO.2011.35.5040 22025146 PMC3675689

[12] Elisei R Schlumberger MJ Müller SP: Cabozantinib in progressive medullary thyroid cancer. *J Clin Oncol.* 2013;31(29):3639–46. 10.1200/JCO.2012.48.4659 24002501 PMC4164813

[13] Le Douarin N Le Lièvre C: [Demonstration of neural origin of calcitonin cells of ultimobranchial body of chick embryo]. *C R Acad Sci Hebd Seances Acad Sci D.* 1970;270(23):2857–60. 4987935

[14] Polak JM Pearse AG Le Lièvre C: Immunocytochemical confirmation of the neural crest origin of avian calcitonin-producing cells. *Histochemistry.* 1974;40(3):209–14. 10.1007/BF00501955 4604566

[15] Kameda Y Nishimaki T Miura M: *Mash1* regulates the development of C cells in mouse thyroid glands. *Dev Dyn.* 2007;236(1):262–70. 10.1002/dvdy.21018 17103415

[16] Johansson E Andersson L Örnros J: Revising the embryonic origin of thyroid C cells in mice and humans. *Development.* 2015;142(20):3519–28. 10.1242/dev.126581 26395490 PMC4631767

[17] Kameda Y: Cellular and molecular events on the development of mammalian thyroid C cells. *Dev Dyn.* 2016;245(3):323–41. 10.1002/dvdy.24377 26661795

[18] Bagheri-Yarmand R Sinha KM Gururaj AE: A novel dual kinase function of the RET proto-oncogene negatively regulates activating transcription factor 4-mediated apoptosis. *J Biol Chem.* 2015;290(18):11749–61. 10.1074/jbc.M114.619833 25795775 PMC4416875

[19] Drosten M Hilken G Böckmann M: Role of MEN2A-derived RET in maintenance and proliferation of medullary thyroid carcinoma. *J Natl Cancer Inst.* 2004;96(16):1231–9. 10.1093/jnci/djh226 15316058

[20] Plaza-Menacho I Barnouin K Barry R: RET functions as a dual-specificity kinase that requires allosteric inputs from juxtamembrane elements. *Cell Rep.* 2016;17(12):3319–32. 10.1016/j.celrep.2016.11.061 28009299 PMC5199340

[21] Read RD Goodfellow PJ Mardis ER: A Drosophila model of multiple endocrine neoplasia type 2. *Genetics.* 2005;171(3):1057–81. 10.1534/genetics.104.038018 15965261 PMC1456812

[22] Cote GJ Grubbs EG Hofmann MC: Thyroid C-cell biology and oncogenic transformation. *Recent Results Cancer Res.* 2015;204:1–39. 10.1007/978-3-319-22542-5_1 26494382 PMC5640981

[23] Barasch JM Mackey H Tamir H: Induction of a neural phenotype in a serotonergic endocrine cell derived from the neural crest. *J Neurosci.* 1987;7(9):2874–83. 3305802 10.1523/JNEUROSCI.07-09-02874.1987 PMC6569149

[24] Moerch U Nielsen HS Lundsgaard D: Flow sorting from organ material by intracellular markers. *Cytometry A.* 2007;71(7):495–500. 10.1002/cyto.a.20418 17542026

[25] Gawade S Mayer C Hafen K: Cell growth dynamics in embryonic and adult mouse thyroid revealed by a novel approach to detect thyroid gland subpopulations. *Thyroid.* 2016;26(4):591–9. 10.1089/thy.2015.0523 26854713

[26] Agrawal N Jiao Y Sausen M: Exomic sequencing of medullary thyroid cancer reveals dominant and mutually exclusive oncogenic mutations in *RET* and *RAS*. *J Clin Endocrinol Metab.* 2013;98(2):E364–9. 10.1210/jc.2012-2703 23264394 PMC3565108

[27] Moura MM Cavaco BM Pinto AE: High prevalence of *RAS* mutations in *RET*-negative sporadic medullary thyroid carcinomas. *J Clin Endocrinol Metab.* 2011;96(5):E863–8. 10.1210/jc.2010-1921 21325462

[28] Schulten H Al-Maghrabi J Al-Ghamdi K: Mutational screening of *RET, HRAS, KRAS, NRAS, BRAF, AKT1*, and *CTNNB1* in medullary thyroid carcinoma. *Anticancer Res.* 2011;31(12):4179–83. 22199277

[29] Boichard A Croux L Al Ghuzlan A: Somatic *RAS* mutations occur in a large proportion of sporadic *RET*-negative medullary thyroid carcinomas and extend to a previously unidentified exon. *J Clin Endocrinol Metab.* 2012;97(10):E2031–5. 10.1210/jc.2012-2092 22865907 PMC3462939

[30] Ciampi R Mian C Fugazzola L: Evidence of a low prevalence of *RAS* mutations in a large medullary thyroid cancer series. *Thyroid.* 2013;23(1):50–7. 10.1089/thy.2012.0207 23240926

[31] Moura MM Cavaco BM Leite V: *RAS* proto-oncogene in medullary thyroid carcinoma. *Endocr Relat Cancer.* 2015;22(5):R235–52. 10.1530/ERC-15-0070 26285815

[32] Grubbs EG Ng PK Bui J: RET fusion as a novel driver of medullary thyroid carcinoma. *J Clin Endocrinol Metab.* 2015;100(3):788–93. 10.1210/jc.2014-4153 25546157 PMC4333032

[33] Ji JH Oh YL Hong M: Identification of driving *ALK* fusion genes and genomic landscape of medullary thyroid cancer. *PLoS Genet.* 2015;11(8):e1005467. 10.1371/journal.pgen.1005467 26295973 PMC4546689

[34] Tomlins SA Rhodes DR Perner S: Recurrent fusion of *TMPRSS2* and ETS transcription factor genes in prostate cancer. *Science.* 2005;310(5748):644–8. 10.1126/science.1117679 16254181

[35] Rubin MA Maher CA Chinnaiyan AM: Common gene rearrangements in prostate cancer. *J Clin Oncol.* 2011;29(27):3659–68. 10.1200/JCO.2011.35.1916 21859993 PMC4874145

[36] Mertens F Johansson B Fioretos T: The emerging complexity of gene fusions in cancer. *Nat Rev Cancer.* 2015;15(6):371–81. 10.1038/nrc3947 25998716

[37] Gottesman MM Lavi O Hall MD: Toward a better understanding of the complexity of cancer drug resistance. *Annu Rev Pharmacol Toxicol.* 2016;56:85–102. 10.1146/annurev-pharmtox-010715-103111 26514196

[38] Diaz LA Jr Williams RT Wu J: The molecular evolution of acquired resistance to targeted EGFR blockade in colorectal cancers. *Nature.* 2012;486(7404):537–40. 10.1038/nature11219 22722843 PMC3436069

[39] Bozic I Gerold JM Nowak MA: Quantifying clonal and subclonal passenger mutations in cancer evolution. *PLoS Comput Biol.* 2016;12(2):e1004731. 10.1371/journal.pcbi.1004731 26828429 PMC4734774

[40] McGranahan N Swanton C: Clonal heterogeneity and tumor evolution: past, present, and the future. *Cell.* 2017;168(4):613–28. 10.1016/j.cell.2017.01.018 28187284

[41] Kantarjian H Giles F Wunderle L: Nilotinib in imatinib-resistant CML and Philadelphia chromosome-positive ALL. *N Engl J Med.* 2006;354(24):2542–51. 10.1056/NEJMoa055104 16775235

[42] de Bono JS Logothetis CJ Molina A: Abiraterone and increased survival in metastatic prostate cancer. *N Engl J Med.* 2011;364(21):1995–2005. 10.1056/NEJMoa1014618 21612468 PMC3471149

[43] Flaherty KT Infante JR Daud A: Combined BRAF and MEK inhibition in melanoma with BRAF V600 mutations. *N Engl J Med.* 2012;367(18):1694–703. 10.1056/NEJMoa1210093 23020132 PMC3549295

[44] Scher HI Fizazi K Saad F: Increased survival with enzalutamide in prostate cancer after chemotherapy. *N Engl J Med.* 2012;367(13):1187–97. 10.1056/NEJMoa1207506 22894553

[45] Zhang C Spevak W Zhang Y: RAF inhibitors that evade paradoxical MAPK pathway activation. *Nature.* 2015;526(7574):583–6. 10.1038/nature14982 26466569

[46] Mok TS Wu YL Ahn MJ: Osimertinib or platinum-pemetrexed in *EGFR* T790M-positive lung cancer. *N Engl J Med.* 2017;376(7):629–40. 10.1056/NEJMoa1612674 27959700 PMC6762027

[47] Carlomagno F Guida T Anaganti S: Disease associated mutations at valine 804 in the RET receptor tyrosine kinase confer resistance to selective kinase inhibitors. *Oncogene.* 2004;23(36):6056–63. 10.1038/sj.onc.1207810 15184865

[48] Mologni L Redaelli S Morandi A: Ponatinib is a potent inhibitor of wild-type and drug-resistant gatekeeper mutant RET kinase. *Mol Cell Endocrinol.* 2013;377(1–2):1–6. 10.1016/j.mce.2013.06.025 23811235

[49] Shah NP Nicoll JM Nagar B: Multiple *BCR-ABL* kinase domain mutations confer polyclonal resistance to the tyrosine kinase inhibitor imatinib (STI571) in chronic phase and blast crisis chronic myeloid leukemia. *Cancer Cell.* 2002;2(2):117–25. 10.1016/S1535-6108(02)00096-X 12204532

[50] Plenker D Riedel M Brägelmann J: Drugging the catalytically inactive state of RET kinase in RET-rearranged tumors. *Sci Transl Med.* 2017;9(394): pii: eaah6144. 10.1126/scitranslmed.aah6144 28615362 PMC5805089

[51] Roberts KG Odell AF Byrnes EM: Resistance to c-KIT kinase inhibitors conferred by V654A mutation. *Mol Cancer Ther.* 2007;6(3):1159–66. 10.1158/1535-7163.MCT-06-0641 17363509

[52] Weil A Martin P Smith R: Pharmacokinetics of vandetanib in subjects with renal or hepatic impairment. *Clin Pharmacokinet.* 2010;49(9):607–18. 10.2165/11534330-000000000-00000 20690783

[53] U.S. Food and Drug Administration: Clinical pharmacology and biometrics review(s). Silver Spring (MD): U.S. Food and Drug Administration;2012 Reference Source

[54] Singh H Brave M Beaver JA: U.S. Food and Drug Administration approval: cabozantinib for the treatment of advanced renal cell carcinoma. *Clin Cancer Res.* 2017;23(2):330–5. 10.1158/1078-0432.CCR-16-1073 27793960

[55] Sherman SI Clary DO Elisei R: Correlative analyses of *RET* and RAS mutations in a phase 3 trial of cabozantinib in patients with progressive, metastatic medullary thyroid cancer. *Cancer.* 2016;122(24):3856–64. 10.1002/cncr.30252 27525386

[56] Schlumberger M Elisei R Müller S: Overall survival analysis of EXAM, a phase III trial of cabozantinib in patients with radiographically progressive medullary thyroid carcinoma. *Ann Oncol.* 2017;28(11):2813–2819. 10.1093/annonc/mdx479 29045520 PMC5834040

[57] Jain S Watson MA DeBenedetti MK: Expression profiles provide insights into early malignant potential and skeletal abnormalities in multiple endocrine neoplasia type 2B syndrome tumors. *Cancer Res.* 2004;64(11):3907–13. 10.1158/0008-5472.CAN-03-3801 15173001

[58] Ameur N Lacroix L Roucan S: Aggressive inherited and sporadic medullary thyroid carcinomas display similar oncogenic pathways. *Endocr Relat Cancer.* 2009;16(4):1261–72. 10.1677/ERC-08-0289 19675075

[59] Maliszewska A Leandro-Garcia LJ Castelblanco E: Differential gene expression of medullary thyroid carcinoma reveals specific markers associated with genetic conditions. *Am J Pathol.* 2013;182(2):350–62. 10.1016/j.ajpath.2012.10.025 23201134

[60] Oczko-Wojciechowska M Swierniak M Krajewska J: Differences in the transcriptome of medullary thyroid cancer regarding the status and type of *RET* gene mutations. *Sci Rep.* 2017;7: 42074. 10.1038/srep42074 28181547 PMC5299608

[61] Songyang Z Carraway KL 3rd Eck MJ: Catalytic specificity of protein-tyrosine kinases is critical for selective signalling. *Nature.* 1995;373(6514):536–9. 10.1038/373536a0 7845468

[62] Gujral TS Singh VK Jia Z: Molecular mechanisms of RET receptor-mediated oncogenesis in multiple endocrine neoplasia 2B. *Cancer Res.* 2006;66(22):10741–9. 10.1158/0008-5472.CAN-06-3329 17108110

[63] Dixit A Torkamani A Schork NJ: Computational modeling of structurally conserved cancer mutations in the RET and MET kinases: the impact on protein structure, dynamics, and stability. *Biophys J.* 2009;96(3):858–74. 10.1016/j.bpj.2008.10.041 19186126 PMC2716637

[64] George Priya Doss C Rajith B Chakraboty C: *In silico* profiling and structural insights of missense mutations in RET protein kinase domain by molecular dynamics and docking approach. *Mol Biosyst.* 2014;10(3):421–36. 10.1039/c3mb70427k 24336963

[65] Yakes FM Chen J Tan J: Cabozantinib (XL184), a novel MET and VEGFR2 inhibitor, simultaneously suppresses metastasis, angiogenesis, and tumor growth. *Mol Cancer Ther.* 2011;10(12):2298–308. 10.1158/1535-7163.MCT-11-0264 21926191

[66] Fujita-Sato S Galeas J Truitt M: Enhanced MET translation and signaling sustains K-Ras-driven proliferation under anchorage-independent growth conditions. *Cancer Res.* 2015;75(14):2851–62. 10.1158/0008-5472.CAN-14-1623 25977330 PMC4506276

[67] Mologni L Gambacorti-Passerini C Goekjian P: RET kinase inhibitors: a review of recent patents (2012–2015). *Expert Opin Ther Pat.* 2017;27(1):91–9. 10.1080/13543776.2017.1238073 27646564

[68] Kodama T Tsukaguchi T Satoh Y: Alectinib shows potent antitumor activity against *RET*-rearranged non-small cell lung cancer. *Mol Cancer Ther.* 2014;13(12):2910–8. 10.1158/1535-7163.MCT-14-0274 25349307

[69] Rahal R Evans EK Hu W: Abstract 2641: the development of potent, selective RET inhibitors that target both wild-type RET and prospectively identified resistance mutations to multi-kinase inhibitors. *Cancer Res.* 2016;76(14):2641 10.1158/1538-7445.AM2016-2641

[70] Brandhuber B Haas J Tuch B: The development of a potent, KDR/VEGFR2-sparing RET kinase inhibitor for treating patients with RET-dependent cancers. *Eur J Cancer.* 2016;69(Supplement 1):S144 10.1016/S0959-8049(16)33028-3

[71] James J Ruggeri B Armstrong RC: CEP-32496: a novel orally active BRAF ^V600E^ inhibitor with selective cellular and *in vivo* antitumor activity. *Mol Cancer Ther.* 2012;11(4):930–41. 10.1158/1535-7163.MCT-11-0645 22319199

[72] Koh YW Shah MH Agarwal K: Sorafenib and Mek inhibition is synergistic in medullary thyroid carcinoma *in vitro*. *Endocr Relat Cancer.* 2012;19(1):29–38. 10.1530/ERC-11-0155 22109971 PMC3717592

[73] Hatzivassiliou G Song K Yen I: RAF inhibitors prime wild-type RAF to activate the MAPK pathway and enhance growth. *Nature.* 2010;464(7287):431–5. 10.1038/nature08833 20130576

[74] Heidorn SJ Milagre C Whittaker S: Kinase-dead BRAF and oncogenic RAS cooperate to drive tumor progression through CRAF. *Cell.* 2010;140(2):209–21. 10.1016/j.cell.2009.12.040 20141835 PMC2872605

[75] Poulikakos PI Zhang C Bollag G: RAF inhibitors transactivate RAF dimers and ERK signalling in cells with wild-type BRAF. *Nature.* 2010;464(7287):427–30. 10.1038/nature08902 20179705 PMC3178447

[76] Li GG Somwar R Joseph J: Antitumor activity of RXDX-105 in multiple cancer types with *RET* rearrangements or mutations. *Clin Cancer Res.* 2017;23(12):2981–90. 10.1158/1078-0432.CCR-16-1887 28011461 PMC5477238

[77] Ohiwa M Murakami H Iwashita T: Characterization of Ret-Shc-Grb2 complex induced by GDNF, MEN 2A, and MEN 2B mutations. *Biochem Biophys Res Commun.* 1997;237(3):747–51. 10.1006/bbrc.1997.7225 9299438

[78] Cox AD Fesik SW Kimmelman AC: Drugging the undruggable RAS: mission possible? *Nat Rev Drug Discov.* 2014;13(11):828–51. 10.1038/nrd4389 25323927 PMC4355017

[79] Stephen AG Esposito D Bagni RK: Dragging ras back in the ring. *Cancer Cell.* 2014;25(3):272–81. 10.1016/j.ccr.2014.02.017 24651010

[80] Papke B Der CJ: Drugging RAS: know the enemy. *Science.* 2017;355(6330):1158–63. 10.1126/science.aam7622 28302824

[81] Goody RS Frech M Wittinghofer A: Affinity of guanine nucleotide binding proteins for their ligands: facts and artefacts. *Trends Biochem Sci.* 1991;16(9):327–8. 10.1016/0968-0004(91)90134-H 1949151

[82] James GL Goldstein JL Brown MS: Polylysine and CVIM sequences of K-RasB dictate specificity of prenylation and confer resistance to benzodiazepine peptidomimetic *in vitro*. *J Biol Chem.* 1995;270(11):6221–6. 10.1074/jbc.270.11.6221 7890759

[83] Rowell CA Kowalczyk JJ Lewis MD: Direct demonstration of geranylgeranylation and farnesylation of Ki-Ras *in vivo*. *J Biol Chem.* 1997;272(22):14093–7. 10.1074/jbc.272.22.14093 9162034

[84] Whyte DB Kirschmeier P Hockenberry TN: K- and N-Ras are geranylgeranylated in cells treated with farnesyl protein transferase inhibitors. *J Biol Chem.* 1997;272(22):14459–64. 10.1074/jbc.272.22.14459 9162087

[85] Grilley-Olson JE Bedard PL Fasolo A: A phase Ib dose-escalation study of the MEK inhibitor trametinib in combination with the PI3K/mTOR inhibitor GSK2126458 in patients with advanced solid tumors. *Invest New Drugs.* 2016;34(6):740–9. 10.1007/s10637-016-0377-0 27450049 PMC7574157

[86] Maurer T Garrenton LS Oh A: Small-molecule ligands bind to a distinct pocket in Ras and inhibit SOS-mediated nucleotide exchange activity. *Proc Natl Acad Sci U S A.* 2012;109(14):5299–304. 10.1073/pnas.1116510109 22431598 PMC3325706

[87] Ostrem JM Peters U Sos ML: K-Ras(G12C) inhibitors allosterically control GTP affinity and effector interactions. *Nature.* 2013;503(7477):548–51. 10.1038/nature12796 24256730 PMC4274051

[88] Zimmermann G Papke B Ismail S: Small molecule inhibition of the KRAS-PDEδ interaction impairs oncogenic KRAS signalling. *Nature.* 2013;497(7451):638–42. 10.1038/nature12205 23698361

[89] Zorde Khvalevsky E Gabai R Rachmut IH: Mutant KRAS is a druggable target for pancreatic cancer. *Proc Natl Acad Sci U S A.* 2013;110(51):20723–8. 10.1073/pnas.1314307110 24297898 PMC3870687

[90] Burns MC Sun Q Daniels RN: Approach for targeting Ras with small molecules that activate SOS-mediated nucleotide exchange. *Proc Natl Acad Sci U S A.* 2014;111(9):3401–6. 10.1073/pnas.1315798111 24550516 PMC3948241

[91] Hunter JC Gurbani D Ficarro SB: *In situ* selectivity profiling and crystal structure of SML-8-73-1, an active site inhibitor of oncogenic K-Ras G12C. *Proc Natl Acad Sci U S A.* 2014;111(24):8895–900. 10.1073/pnas.1404639111 24889603 PMC4066474

[92] Lim SM Westover KD Ficarro SB: Therapeutic targeting of oncogenic K-Ras by a covalent catalytic site inhibitor. *Angew Chem Int Ed Engl.* 2014;53(1):199–204. 10.1002/anie.201307387 24259466 PMC3914205

[93] Upadhyaya P Qian Z Selner NG: Inhibition of Ras signaling by blocking Ras-effector interactions with cyclic peptides. *Angew Chem Int Ed Engl.* 2015;54(26):7602–6. 10.1002/anie.201502763 25950772 PMC4591930

[94] Athuluri-Divakar SK Vasquez-Del Carpio R Dutta K: A small molecule RAS-mimetic disrupts RAS association with effector proteins to block signaling. *Cell.* 2016;165(3):643–55. 10.1016/j.cell.2016.03.045 27104980 PMC5006944

[95] Lito P Solomon M Li LS: Allele-specific inhibitors inactivate mutant KRAS G12C by a trapping mechanism. *Science.* 2016;351(6273):604–8. 10.1126/science.aad6204 26841430 PMC4955282

[96] Lu S Jang H Gu S: Drugging Ras GTPase: a comprehensive mechanistic and signaling structural view. *Chem Soc Rev.* 2016;45(18):4929–52. 10.1039/c5cs00911a 27396271 PMC5021603

[97] McCormick F: K-Ras protein as a drug target. *J Mol Med (Berl).* 2016;94(3):253–8. 10.1007/s00109-016-1382-7 26960760 PMC4803814

[98] Ostrem JM Shokat KM: Direct small-molecule inhibitors of KRAS: from structural insights to mechanism-based design. *Nat Rev Drug Discov.* 2016;15(11):771–85. 10.1038/nrd.2016.139 27469033

[99] Papke B Murarka S Vogel HA: Identification of pyrazolopyridazinones as PDEδ inhibitors. *Nat Commun.* 2016;7: 11360. 10.1038/ncomms11360 27094677 PMC4843002

[100] Patricelli MP Janes MR Li LS: Selective inhibition of oncogenic KRAS output with small molecules targeting the inactive state. *Cancer Discov.* 2016;6(3):316–29. 10.1158/2159-8290.CD-15-1105 26739882

[101] Cetin M Evenson WE Gross GG: RasIns: genetically encoded intrabodies of activated Ras proteins. *J Mol Biol.* 2017;429(4):562–73. 10.1016/j.jmb.2016.11.008 27865780 PMC5798998

[102] Kauke MJ Traxlmayr MW Parker JA: An engineered protein antagonist of K-Ras/B-Raf interaction. *Sci Rep.* 2017;7(1): 5831. 10.1038/s41598-017-05889-7 28724936 PMC5517481

[103] Keeton AB Salter EA Piazza GA: The RAS-effector interaction as a drug target. *Cancer Res.* 2017;77(2):221–6. 10.1158/0008-5472.CAN-16-0938 28062402 PMC5243175

[104] Martín-Gago P Fansa EK Klein CH: A PDE6δ-KRas inhibitor chemotype with up to seven H-Bonds and picomolar affinity that prevents efficient inhibitor release by Arl2. *Angew Chem Int Ed Engl.* 2017;56(9):2423–8. 10.1002/anie.201610957 28106325

[105] Martín-Gago P Fansa EK Winzker M: Covalent protein labeling at glutamic acids. *Cell Chem Biol.* 2017;24(5):589–597.e5. 10.1016/j.chembiol.2017.03.015 28434875

[106] Novotny CJ Hamilton GL McCormick F: Farnesyltransferase-Mediated Delivery of a Covalent Inhibitor Overcomes Alternative Prenylation to Mislocalize K-Ras. *ACS Chem Biol.* 2017;12(7):1956–62. 10.1021/acschembio.7b00374 28530791 PMC6070134

[107] Ross SJ Revenko AS Hanson LL: Targeting KRAS-dependent tumors with AZD4785, a high-affinity therapeutic antisense oligonucleotide inhibitor of KRAS. *Sci Transl Med.* 2017;9(394): pii: eaal5253. 10.1126/scitranslmed.aal5253 28615361

[108] Sakamoto K Kamada Y Sameshima T: K-Ras(G12D)-selective inhibitory peptides generated by random peptide T7 phage display technology. *Biochem Biophys Res Commun.* 2017;484(3):605–11. 10.1016/j.bbrc.2017.01.147 28153726

[109] Spencer-Smith R Koide A Zhou Y: Inhibition of RAS function through targeting an allosteric regulatory site. *Nat Chem Biol.* 2017;13(1):62–8. 10.1038/nchembio.2231 27820802 PMC5193369

[110] Welsch ME Kaplan A Chambers JM: Multivalent small-molecule pan-RAS inhibitors. *Cell.* 2017;168(5):878–889.e29. 10.1016/j.cell.2017.02.006 28235199 PMC5362268

[111] Chandra A Grecco HE Pisupati V: The GDI-like solubilizing factor PDEδ sustains the spatial organization and signalling of Ras family proteins. *Nat Cell Biol.* 2011;14(2):148–58. 10.1038/ncb2394 22179043

[112] Hong DS Cabanillas ME Wheler J: Inhibition of the Ras/Raf/MEK/ERK and RET kinase pathways with the combination of the multikinase inhibitor sorafenib and the farnesyltransferase inhibitor tipifarnib in medullary and differentiated thyroid malignancies. *J Clin Endocrinol Metab.* 2011;96(4):997–1005. 10.1210/jc.2010-1899 21289252 PMC3070247

[113] Lam ET Ringel MD Kloos RT: Phase II clinical trial of sorafenib in metastatic medullary thyroid cancer. *J Clin Oncol.* 2010;28(14):2323–30. 10.1200/JCO.2009.25.0068 20368568 PMC2881718

[114] Brahmer JR Tykodi SS Chow LQ: Safety and activity of anti-PD-L1 antibody in patients with advanced cancer. *N Engl J Med.* 2012;366(26):2455–65. 10.1056/NEJMoa1200694 22658128 PMC3563263

[115] Topalian SL Hodi FS Brahmer JR: Safety, activity, and immune correlates of anti-PD-1 antibody in cancer. *N Engl J Med.* 2012;366(26):2443–54. 10.1056/NEJMoa1200690 22658127 PMC3544539

[116] Couzin-Frankel J: Breakthrough of the year 2013. Cancer immunotherapy. *Science.* 2013;342(6165):1432–3. 10.1126/science.342.6165.1432 24357284

[117] Sharma P Allison JP: The future of immune checkpoint therapy. *Science.* 2015;348(6230):56–61. 10.1126/science.aaa8172 25838373

[118] Chen DS Mellman I: Elements of cancer immunity and the cancer-immune set point. *Nature.* 2017;541(7637):321–30. 10.1038/nature21349 28102259

[119] French JD Bible K Spitzweg C: Leveraging the immune system to treat advanced thyroid cancers. *Lancet Diabetes Endocrinol.* 2017;5(6):469–81. 10.1016/S2213-8587(16)30277-7 27773653

[120] Bongiovanni M Rebecchini C Saglietti C: Very low expression of PD-L1 in medullary thyroid carcinoma. *Endocr Relat Cancer.* 2017;24(6):L35–L38. 10.1530/ERC-17-0104 28420659 PMC5457503

[121] Herbst RS Soria JC Kowanetz M: Predictive correlates of response to the anti-PD-L1 antibody MPDL3280A in cancer patients. *Nature.* 2014;515(7528):563–7. 10.1038/nature14011 25428504 PMC4836193

[122] Rizvi NA Hellmann MD Snyder A: Cancer immunology. Mutational landscape determines sensitivity to PD-1 blockade in non-small cell lung cancer. *Science.* 2015;348(6230):124–8. 10.1126/science.aaa1348 25765070 PMC4993154

[123] Schott M Feldkamp J Klucken M: Calcitonin-specific antitumor immunity in medullary thyroid carcinoma following dendritic cell vaccination. *Cancer Immunol Immunother.* 2002;51(11–12):663–8. 10.1007/s00262-002-0325-z 12439612 PMC11032831

[124] Papewalis C Wuttke M Seissler J: Dendritic cell vaccination with xenogenic polypeptide hormone induces tumor rejection in neuroendocrine cancer. *Clin Cancer Res.* 2008;14(13):4298–305. 10.1158/1078-0432.CCR-08-0587 18594013

[125] Wuttke M Papewalis C Meyer Y: Amino acid-modified calcitonin immunization induces tumor epitope-specific immunity in a transgenic mouse model for medullary thyroid carcinoma. *Endocrinology.* 2008;149(11):5627–34. 10.1210/en.2008-0631 18617610

[126] Bachleitner-Hofmann T Friedl J Hassler M: Pilot trial of autologous dendritic cells loaded with tumor lysate(s) from allogeneic tumor cell lines in patients with metastatic medullary thyroid carcinoma. *Oncol Rep.* 2009;21(6):1585–92. 10.3892/or_00000391 19424640

[127] Kalos M Levine BL Porter DL: T cells with chimeric antigen receptors have potent antitumor effects and can establish memory in patients with advanced leukemia. *Sci Transl Med.* 2011;3(95):95ra73. 10.1126/scitranslmed.3002842 21832238 PMC3393096

[128] Rosenberg SA Restifo NP: Adoptive cell transfer as personalized immunotherapy for human cancer. *Science.* 2015;348(6230):62–8. 10.1126/science.aaa4967 25838374 PMC6295668

[129] Porter DL Hwang W Frey NV: Chimeric antigen receptor T cells persist and induce sustained remissions in relapsed refractory chronic lymphocytic leukemia. *Sci Transl Med.* 2015;7(303):303ra139. 10.1126/scitranslmed.aac5415 26333935 PMC5909068

[130] Johnson LA June CH: Driving gene-engineered T cell immunotherapy of cancer. *Cell Res.* 2017;27(1):38–58. 10.1038/cr.2016.154 28025979 PMC5223234

[131] NetMHC 3.4. Reference Source

[132] Plaza-Menacho I Burzynski GM de Groot JW: Current concepts in RET-related genetics, signaling and therapeutics. *Trends Genet.* 2006;22(11):627–36. 10.1016/j.tig.2006.09.005 16979782

[133] Runeberg-Roos P Saarma M: Neurotrophic factor receptor RET: structure, cell biology, and inherited diseases. *Ann Med.* 2007;39(8):572–80. 10.1080/07853890701646256 17934909

[134] Linette GP Stadtmauer EA Maus MV: Cardiovascular toxicity and titin cross-reactivity of affinity-enhanced T cells in myeloma and melanoma. *Blood.* 2013;122(6):863–71. 10.1182/blood-2013-03-490565 23770775 PMC3743463

[135] Morgan RA Chinnasamy N Abate-Daga D: Cancer regression and neurological toxicity following anti-MAGE-A3 TCR gene therapy. *J Immunother.* 2013;36(2):133–51. 10.1097/CJI.0b013e3182829903 23377668 PMC3581823

[136] Wang QJ Yu Z Griffith K: Identification of T-cell receptors targeting KRAS-mutated human tumors. *Cancer Immunol Res.* 2016;4(3):204–14. 10.1158/2326-6066.CIR-15-0188 26701267 PMC4775432

[137] Saunders LR Bankovich AJ Anderson WC: A DLL3-targeted antibody-drug conjugate eradicates high-grade pulmonary neuroendocrine tumor-initiating cells *in vivo*. *Sci Transl Med.* 2015;7(302):302ra136. 10.1126/scitranslmed.aac9459 26311731 PMC4934375

[138] Saunders LR Williams SA Bheddah S: Abstract 3093: expression of DLL3 in metastatic melanoma, glioblastoma and high-grade extrapulmonary neuroendocrine carcinomas as potential indications for rovalpituzumab tesirine (Rova-T; SC16LD6.5), a delta-like protein 3 (DLL3)-targeted antibody drug conjugate (ADC). *Proc Amer Assoc for Cancer Res.* 2017;7(13 Suppl): Abstract 3093. 10.1158/1538-7445.AM2017-3093

[139] Rudin CM Pietanza MC Bauer TM: Rovalpituzumab tesirine, a DLL3-targeted antibody-drug conjugate, in recurrent small-cell lung cancer: a first-in-human, first-in-class, open-label, phase 1 study. *Lancet Oncol.* 2017;18(1):42–51. 10.1016/S1470-2045(16)30565-4 27932068 PMC5481162

[140] Shin SM Choi DK Jung K: Antibody targeting intracellular oncogenic Ras mutants exerts anti-tumour effects after systemic administration. *Nat Commun.* 2017;8:15090. 10.1038/ncomms15090 28489072 PMC5436137

[141] Strosberg J El-Haddad G Wolin E: Phase 3 Trial of ^177^Lu-dotatate for midgut neuroendocrine tumors. *N Engl J Med.* 2017;376(2):125–35. 10.1056/NEJMoa1607427 28076709 PMC5895095

[142] Waldherr C Schumacher T Pless M: Radiopeptide transmitted internal irradiation of non-iodophil thyroid cancer and conventionally untreatable medullary thyroid cancer using. *Nucl Med Commun.* 2001;22(6):673–8. 10.1097/00006231-200106000-00011 11403179

[143] Bodei L Handkiewicz-Junak D Grana C: Receptor radionuclide therapy with 90Y-DOTATOC in patients with medullary thyroid carcinomas. *Cancer Biother Radiopharm.* 2004;19(1):65–71. 10.1089/108497804773391694 15068613

[144] Makis W McCann K McEwan AJ: Medullary thyroid carcinoma (MTC) treated with 177Lu-DOTATATE PRRT: a report of two cases. *Clin Nucl Med.* 2015;40(5):408–12. 10.1097/RLU.0000000000000706 25674858

[145] Vaisman F Rosado de Castro PH Lopes FP: Is there a role for peptide receptor radionuclide therapy in medullary thyroid cancer? *Clin Nucl Med.* 2015;40(2):123–7. 10.1097/RLU.0000000000000628 25546220

[146] Salavati A Puranik A Kulkarni HR: Peptide receptor radionuclide therapy (PRRT) of medullary and nonmedullary thyroid cancer using radiolabeled somatostatin analogues. *Semin Nucl Med.* 2016;46(3):215–24. 10.1053/j.semnuclmed.2016.01.010 27067502

[147] Conry BG Papathanasiou ND Prakash V: Comparison of ^68^Ga-DOTATATE and ^18^F-fluorodeoxyglucose PET/CT in the detection of recurrent medullary thyroid carcinoma. *Eur J Nucl Med Mol Imaging.* 2010;37(1):49–57. 10.1007/s00259-009-1204-z 19662413

[148] Treglia G Castaldi P Villani MF: Comparison of 18F-DOPA, ^18^F-FDG and ^68^Ga-somatostatin analogue PET/CT in patients with recurrent medullary thyroid carcinoma. *Eur J Nucl Med Mol Imaging.* 2012;39(4):569–80. 10.1007/s00259-011-2031-6 22223169

[149] Ozkan ZG Kuyumcu S Uzum AK: Comparison of ⁶⁸Ga-DOTATATE PET-CT, ¹⁸F-FDG PET-CT and 99mTc-(V)DMSA scintigraphy in the detection of recurrent or metastatic medullary thyroid carcinoma. *Nucl Med Commun.* 2015;36(3):242–50. 10.1097/MNM.0000000000000240 25369749

[150] Tran K Khan S Taghizadehasl M: Gallium-68 Dotatate PET/CT is superior to other imaging modalities in the detection of medullary carcinoma of the thyroid in the presence of high serum calcitonin. *Hell J Nucl Med.* 2015;18(1):19–24. 25679074 10.1967/s002449910163

[151] Herac M Niederle B Raderer M: Expression of somatostatin receptor 2A in medullary thyroid carcinoma is associated with lymph node metastasis. *APMIS.* 2016;124(10):839–45. 10.1111/apm.12584 27539746

[152] Papotti M Kumar U Volante M: Immunohistochemical detection of somatostatin receptor types 1–5 in medullary carcinoma of the thyroid. *Clin Endocrinol (Oxf).* 2001;54(5):641–9. 10.1046/j.1365-2265.2001.01175.x 11380495

